# Interactive effects of compost and pre-planting soil moisture on plant biomass, nutrition and formation of mycorrhizas: a context dependent response

**DOI:** 10.1038/s41598-017-18780-2

**Published:** 2018-01-24

**Authors:** H. T. T. Ngo, T. R. Cavagnaro

**Affiliations:** The Waite Research Institute, and The School of Agriculture, Food and Wine, The University of Adelaide, The Waite Campus, PMB 1 Glen Osmond, Adelaide, South Australia 5064 Australia

## Abstract

We aimed to investigate the combined impacts of compost addition and pre-planting soil moisture conditions, on plant-available nutrients, and subsequent impacts on the biomass, nutrition and formation of AM by two important crop species. A glasshouse study was undertaken in which wheat and tomato plants were grown in compost amended or un-amended soil that was subjected to different moisture regimes prior to planting. The availability of P was strongly influenced by compost addition, but not pre-planting moisture conditions. In contrast, mineral N pools were affected by compost addition and pre-planting soil moisture conditions in complex ways. These changes in nutrient availability affected plant biomass, nutrient uptake and formation of AM. In general, plant performance was better where pre-planting soil moisture conditions were wet or dry, and worse where they involved a wet/dry cycle, and mycorrhizal colonisation was lower where compost was added to the soil. That pre-planting moisture conditions affect the biomass of subsequent crops is an important finding, the potential implications of which are considered here.

## Introduction

As the climate becomes more variable, so too will soil moisture^1–3^. Moisture is a major driver of organic matter decomposition and mineralisation in soil. Not only does the amount of water in the soil affect organic matter decomposition and mineralisation, but so to do the temporal dynamics of soil moisture. For example, nutrient release from organic matter and nutrient cycling processes are strongly affected by wetting/drying cycles in soil^4–9^.

Soil moisture conditions prior to planting, can have an impact on the subsequent growth and nutrition of plants, via impacts on soil nutrient availability at the time of planting^10^. Impacts of soil moisture on nutrient availability and cycling may be especially important for systems where organic matter is a dominant source of nutrients. This is because the cycling and release of nutrients from organic matter sources is largely a microbially-mediated process, and the activity of soil microbes is strongly moisture dependent^8,11^. Further, it has been found that the formation and functioning of arbuscular mycorrhizas (AM) - associations between plant roots and a specialised group of soil fungi that can play an important role in plant nutrient acquisition – can be affected by pre-planting soil moisture conditions, presumably due to impacts on soil nutrients^10^. The formation of AM is also affected by soil nutrient status, with reductions in colonisation of roots by arbuscular mycorrhizal fungi (AMF) reported with increasing soil P and N supply^12,13^.

Composts have long been used to deliver nutrients to plants^14–16^, and generally do not adversely affect the formation of AM see^17^, for recent review. When incorporated into soil, compost is mineralised, thereby providing a sustained release of available nutrients to plants^18–21^. While it is well established that the cycling of nutrients is affected by soil moisture^11^, there has to our knowledge, been no work to investigate the impact of pre-planting soil moisture conditions on the release of nutrients from composts, and the subsequent (and concurrent) impacts on plant biomass, nutrition and formation of AM.

Here we present results of a study in which we sought to investigate the combined impacts of compost addition and pre-planting soil moisture conditions, on plant-available nutrients, and the biomass, nutrition and mycorrhizal colonisation of two important crop species, wheat and tomato. The experiment included moisture treatments that were wet, dry, or cycled between wet and dry conditions, in order to investigate impacts of variable water supply on compost mineralisation and the subsequent consequences for plants.

## Results

### Soil nutrients

**Figure 1:**
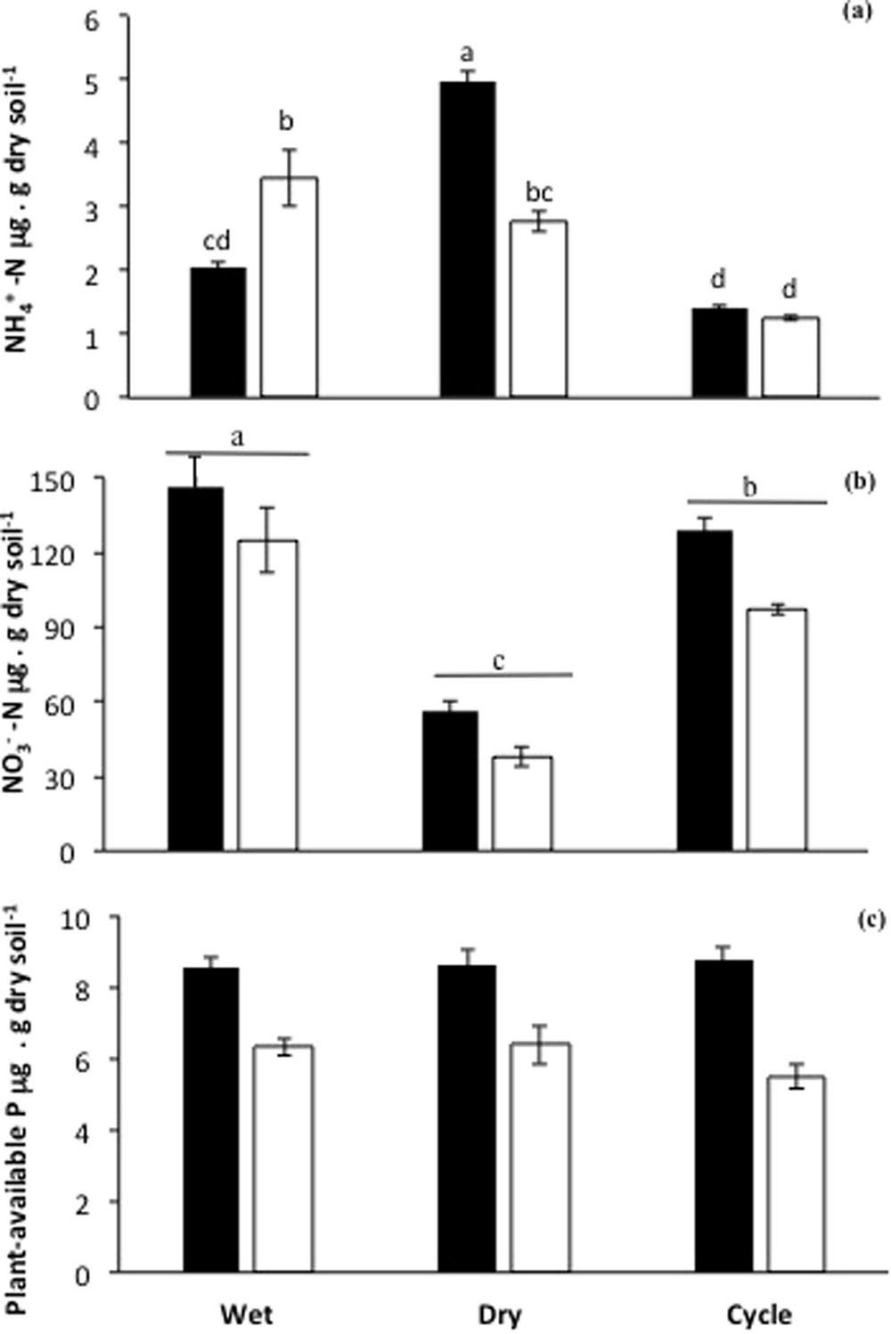
Concentrations of (**a**) NH_4_^+^-N, (**b**) NO_3_^−^-N, and (**c**) plant-available (Colwell) P, in soils at the time of planting, that were amended with compost (black bars), or were un-amended (white bars), and subjected to different pre-planting soil moisture conditions (see text). Values are means ± SE (n = 10). Means followed by the same letters are not significantly different at the P < 0.05 level.

Pre-planting watering treatments had a significant impact on soil physicochemical properties measured at the time of planting (Fig. 1). For NH_4_^+^-N (Fig. 1a) there was a significant (P < 0.0001) interaction between pre-planting soil moisture treatment and compost addition treatment. Specifically, the concentration of NH_4_^+^-N (Fig. 1a) was lowest in soil from the cycle treatment, and did not differ between compost-amended and un-amended treatments. In contrast, in the wet treatment, the concentration of NH_4_^+^-N was significantly higher where no compost was added to the soil. The reverse was true in the dry treatment where the concentration of NH_4_^+^-N was higher in the soil with compost (and highest overall) than without compost.

For NO_3_^−^-N at the time of planting (Fig. 1b), the interaction between pre-planting soil moisture treatment and compost addition treatment was not significant (P > 0.05), but the main effects of compost addition treatment and pre-planting soil moisture treatments were. Specifically, the concentration of NO_3_^−^-N was significantly (P < 0.001) higher with compost addition, irrespective of soil moisture treatment. Further, soil NO_3_^−^-N concentration differed significantly (P < 0.0001) among soil moisture treatments, irrespective of compost addition, in the order Dry < Cycle < Wet.

For plant-available P at the time of planting (Fig. 1c), neither the interaction nor the main effect of soil moisture treatment were significant (P > 0.05). However, plant-available (Colwell) P was significantly (P < 0.0001) higher with compost-amendment, irrespective of pre-planting soil moisture treatment.

### Plant biomass and nutrition: tomato and wheat

**Figure 2:**
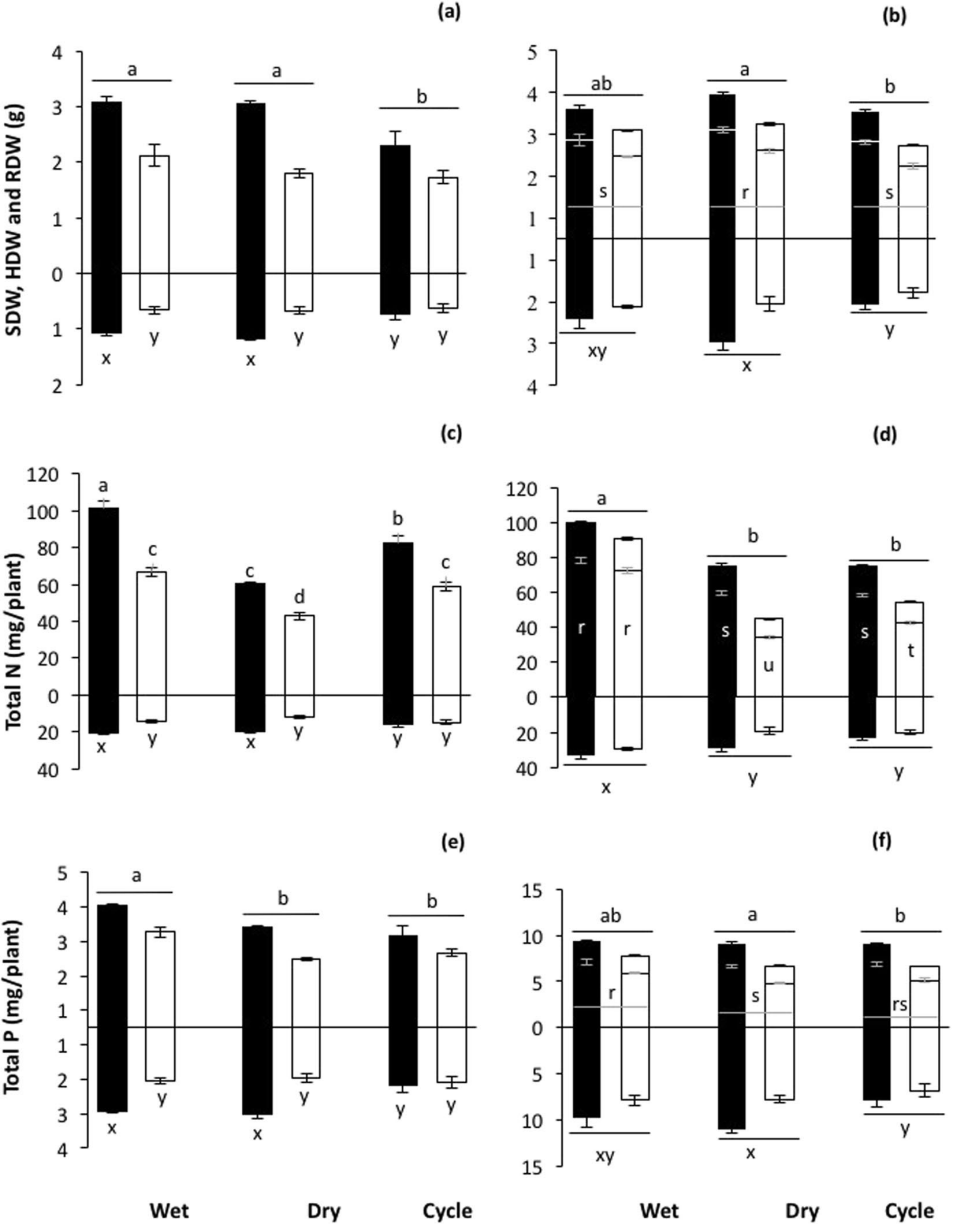
Biomass of (**a**) tomato and (**b**) wheat; N content of (**c**) tomato and (**d**) wheat; and P content of (**e**) tomato and (**f**) wheat, grown in soils that were amended with compost (black bars), or were un-amended (white bars), and subjected to different pre-planting soil moisture conditions (see text). Values, where are means ± S.E. (n = 5), for above- and below-ground tissues are presented above- and below the x-axis, respectively. For tomatoes, above-ground biomass was for vegetative tissues only. For wheat, above-ground biomass was divided into shoots and heads, which presented as the lower and upper portions of the bars above the x-axis, respectively. Where the ANOVA revealed a significant two-way interaction bars are labelled with individual letters; means followed by the same letter are not significantly different at the P < 0.05 level. Where the main effect of compost was significant this is reported in the text. Where the main effect of pre-planting watering treatment was significant, differences between watering treatments (i.e. pooled over compost treatment within a given compost watering treatment) are indicated with letters sitting immediately above a horizontal line. N.B. valid statistical comparisons cannot be made between plant species, nor tissue types.

The biomass of tomato plants was significantly affected by compost addition and pre-planting moisture conditions (Fig. 2a). Specifically, shoot dry weights were significantly (P < 0.0001) higher in compost-amended soil than un-amended soil. Similarly, pre-planting moisture conditions also significantly (P < 0.01) affected tomato shoot dry weight, irrespective of compost addition treatment, with shoot dry weights in the pre-planting wet treatment similar to those in the pre-planting dry treatment, and higher than the pre-planting cycle treatment. To further explore these data, targeted t-tests were performed to compare between compost addition treatments within each of the pre-planting moisture addition treatments separately. This analysis revealed greater shoot dry weight with compost addition in the dry and wet treatments, but not the cycle treatment. For tomato root dry weights, there was a significant (P < 0.05) interaction between pre-planting moisture conditions and compost addition treatment. Specifically, relative to the un-amended treatments, tomato root dry weights were higher with compost addition in the pre-planting wet and dry treatments, but not the pre-planting cycle treatment.

The biomass of wheat was significantly affected by compost addition and pre-planting moisture conditions (Fig. 2b). Irrespective of pre-planting soil moisture treatment, the biomass of wheat was greater in compost-amended soils than un-amended soils; specifically, wheat heads (P < 0.0001), wheat shoots (P < 0.0001) and wheat roots (P < 0.001). Pre-planting soil moisture conditions, irrespective of compost addition treatment, also had a significant impact on shoot (P < 0.001), head (P < 0.01) and root (P < 0.01) dry weights. Specifically, shoot and head dry weights were highest in the pre-planting dry treatment, intermediate in the pre-planting wet treatment, and lowest in the pre-planting cycle treatment.

Compost addition and pre-planting moisture conditions altered the total N in tomato (Fig. 2c) and wheat (Fig. 2d). In tomato, the total N in both shoots and roots was modulated by the two-way interaction between compost addition and pre-planting moisture conditions (P < 0.05 and P < 0.01, respectively). Total N in tomato shoots was highest in the pre-planting wet treatment with compost, followed by the pre-planting cycle and dry treatments with compost, respectively. The total N in tomato shoots in the pre-planting wet and cycle treatments without compost was lower than where compost was added, but higher than in the pre-planting dry treatment without compost, which was lowest of all. In contrast to the shoots, total N in roots was significantly higher in the pre-planting wet and dry treatments with compost amendment compared to the other treatments. For total N in the shoots of wheat (Fig. 2d), there was a significant two-way interaction (P < 0.0001); specifically, total N in the pre-planting wet treatment was highest in both compost-amended and un-amended soil, and in the pre-planting dry and cycle treatments total N was higher with compost addition, but lower than in the pre-planting wet treatment. For total N in the heads and roots of wheat, only the main effects were significant. Specifically, total N was higher with compost addition than without, irrespective of pre-planting moisture treatment (heads: P < 0.0001; roots: P < 0.001), and highest in the pre-planting wet treatment, irrespective of compost addition (heads: P < 0.0001; roots: P < 0.0001).

Plant P uptake, measured as P contents, were affected by the experimental treatments. For tomato (Fig. 2e), total P in shoots was significantly higher in the treatments with compost than without compost (P < 0.0001). Further, total P was highest, irrespective of compost addition treatment, in the wet pre-planting moisture treatment (P < 0.0001). For total P in tomato roots, there was a significant two-way interaction (P < 0.01); total P in roots was highest in the pre-planting wet and dry treatments with compost addition compared to all other treatments. Total P in the wheat (Fig. 2f) heads, shoots and roots was higher in compost-amended soils than in un-amended soils (heads: P < 0.0001; shoots: P < 0.0001; roots: P = 0.001, respectively). Further, total P in the wheat shoots, irrespective of compost addition treatment, was highest, intermediate and lowest in the pre-planting wet, cycle and dry treatments, respectively (P < 0.01). In contrast, the total P in wheat heads, irrespective of compost addition treatment, was highest, intermediate and lowest in the pre-planting dry, wet and cycle treatments, respectively (P < 0.05). Total P in wheat roots followed the same trend as wheat heads (P < 0.05).

### Formation of mycorrhizas in tomato and wheat

**Figure 3:**
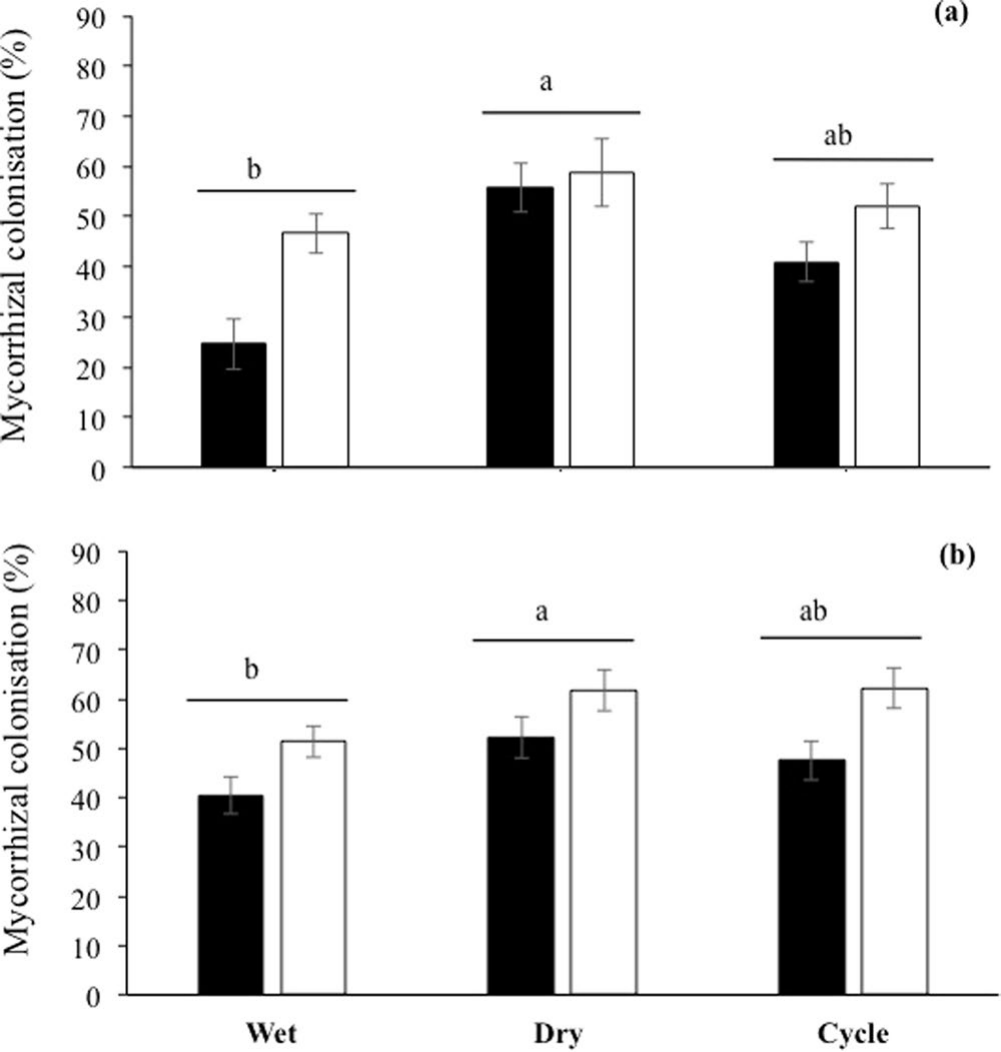
Mycorrhizal colonisation (precent root length colonised) of (**a**) tomato and (**b**) wheat, grown in soils that had been amended with compost (black bars), or were un-amended (white bars), and subjected to different pre-planting soil moisture conditions (see text). Values are means ± SE (n = 5). Means followed by the same letters are not significantly different at the P < 0.05 level. N.B. valid statistical comparisons cannot be made between plant species.

The formation of mycorrhizas by both tomato and wheat was affected by compost addition and pre-planting moisture conditions. Percent mycorrhizal colonisation of tomato (Fig. 3a) and wheat (Fig. 3b) roots was significantly lower, irrespective of pre-planting soil moisture treatment, in the soils where compost was added (P < 0.01 and P < 0.01, respectively). In terms of pre-planting moisture impacts, irrespective of compost addition, percent AM colonisation in tomato was significantly higher in the pre-planting dry treatment than in the pre-planting wet treatment, with those in the cycle treatment intermediate (P < 0.001). For wheat, the pattern of changes in percent mycorrhizal colonisation due to pre-planting moisture conditions were similar to those of tomato (P < 0.05).

## Discussion

The addition of compost to the soil, and soil moisture regimes prior to planting, affected soil nutrient levels at the time of planting. Generally, soil nutrient contents, and plant biomass and nutrient contents, were higher with compost addition, and AM colonisation was lower. Responses to pre-planting soil moisture conditions were also observed, with a clear indication that soil moisture conditions prior to planting can have a major impact on soil nutrients, and subsequent plant biomass, nutrition and the formation of AM. The reasons underlying these impacts and their potential implications are now discussed.

### Soil nutrients

Concentrations of NH_4_^+^-N in the soil at the time of planting were generally low, consistent with earlier work^10^. Overall, NH_4_^+^-N concentrations at the time of planting were highest in soils in the dry treatment, and lowest in the wet and cycle treatments. However, these differences between treatments were small (approx. 2 mg. kg^−1^) compared to the changes in NO_3_^−^-N that were observed. Soil NO_3_^−^-N concentrations at the time of planting were high; NO_3_^−^-N concentrations were higher with compost addition than without, and were substantially higher in soils in the wet and cycle treatments than when pre-planting conditions were dry. The higher levels of NO_3_^−^-N in the compost-amended soils suggest that N in the compost was mineralised to NH_4_^+^-N and in turn nitrified to NO_3_^−^N, as was expected. The large build-up of NO_3_^−^-N, but not NH_4_^+^-N, especially in the wet treatment, suggests that soil moisture was sufficiently high in the wet treatments to stimulate nitrification, for example due to enhanced substrate diffusion, increased microbial activity and/or the abundance of nitrifiers in the soil, but no so moist as to favour denitrification^22–24^. In the cycle treatment, NO_3_^−^-N concentrations were not as high as in the wet treatment. This may be because the N mineralisation flush after rewetting soil did not compensate for the mineralisation decline during the drying period^1^. Together, these changes in mineral N pools due to interactive effects of soil moisture and compost addition prior to planting may have an important impact on plant biomass and the formation of AM.

Plant-available P in the soils was higher with compost addition at the time of planting, as expected. Interestingly, there was little difference in plant-available P between pre-planting soil moisture treatments. This lack of an interactive effect is in contrast with earlier work, indicating an increase in plant-available P after rewetting soils^25^, and in agreement with others indicating only a minor impact of soil moisture on P mineralisation in soils^26,27^.

### Plant biomass and nutrition

The addition of compost to the soil resulted greater biomass and nutrient uptake by both tomato and wheat, compared to where compost was not added to the soil. The concentration of plant-available P in the soil at the time of planting was low, and increased with compost addition. Thus, it is likely that the improvements in biomass associated with compost addition were in part due to soil P amelioration^28–30^. In contrast, impacts of compost addition on mineral N pools in the soil were less consistent. Given the highly dynamic nature of soil N cycling^8^, and the fact that it is strongly influenced by soil moisture^11^, this was not unexpected.

Pre-planting soil moisture conditions had a significant impact on the subsequent biomass and nutrient uptake by both tomato and wheat. Although responses were less straightforward than for compost addition, plant performance was generally poorer where pre-planting soil moisture conditions involved a wet/dry cycle. This difference in biomass between treatments cannot be easily explained by concomitant changes in soil nutrients, which are likely associated with moisture effects of microbialy-mediated nutrient mineralisation, but some insights can be gained, as follows.

Where the pre-planting conditions were dry, the biomass of tomato and wheat was greatest, despite the fact that available N was lowest and plant-available P was similar to that in the other pre-planting watering treatments. This suggests that these plants were not N-limited, and that the greater biomass cannot be attributed to P supply alone. In contrast, where pre-planting soil moisture was cycled rather than held constant, the biomass of tomato and wheat was lowest and so to was the total P content of the plants (i.e. heads, shoots and roots together) but total N was not. This suggests that P, but not N, was more limiting to the biomass of tomato and wheat in this situation. Finally, when tomato was grown in soil where pre-planting conditions were wet, P contents were high but biomass was not different to that where plants were grown in soil where pre-planting conditions were dry, and plant P contents were low. Thus, it may be that the soil was deficient in other nutrients^31,32^. Together, these examples suggest that impacts of pre-planting soil moisture conditions on subsequent plant biomass are complex. It is likely that these impacts extend beyond factors measured here, and future work should seek to investigate impacts on other soil nutrients, and/or soil microbial communities involved in their cycling and release.

Mycorrhizal colonisation of both wheat and tomato was affected by compost addition and pre-planting soil moisture conditions. AM colonisation was significantly lower with compost addition, irrespective of pre-planting watering treatment. Although there are many studies where AM colonisation was not reduced following compost addition see^17^, for recent review, the increase in soil P with compost addition observed here, and the fact that AM colonisation is generally decreased with P addition^12,33^, help to explain this reduction. AM colonisation was also affected by pre-planting moisture conditions, irrespective of compost addition; colonisation was lower where pre-planting conditions were wet compared to when they were dry. These differences may be due to changes in the inoculum potential of the soil^34^, whereby under wet pre-planting conditions propagules of AMF may have germinated, but failed to locate a host plant^35^. These propagules may then have been unable to colonise a host plant when one was introduced. Although not the focus on this study, impacts of the experimental treatments of the functioning of AM are also of interest. Similarly, it would also be interesting to quantify impacts of pre-planting moisture conditions on the full range of AM propagules in future work.

## Conclusions

This study clearly demonstrates that pre-planting soil moisture conditions and compost addition interactively affect soil nutrients. This in turn had consequences for plant biomass and nutrition and the formation of AM. It is likely that the carry-over (or soil moisture legacy) effects on plant biomass, nutrition and mycorrhizal colonisation, were mediated by both N and P availability in the soil, and plant N and P contents. While not investigated here, growth of the extraradical hyphae of AMF, and their interaction with microbes involved in mineralisation^36^, is worthy of further consideration. Similarly, changes in soil microbial communities, with an emphasis on microbes involved in nutrient mineralisation, will also be important. Putting the underlying mechanisms involved to one side, this is an important finding; it highlights the impact and importance of context dependency in experiments. Although not often taken into consideration, these carry-over effects are worthy of further consideration both in a practical and experimental sense. For example, the somewhat reduced biomass of plants grown in soil where pre-planting soil moisture was cycled, may have important implications in the context of increased climate variability with climate change. This, however is speculative and requires further investigation.

## Methods

### Overview

This experiment involved subjecting compost amended or un-amended soil to different pre-planting soil moisture regimes (wet, dry, or cycled) for a period of 40 days. After this pre-planting phase, water was added to all soils so that they were at the same moisture content (wet), and tomato and wheat plants grown. This experiment, which is explained in more detail below, was designed to investigate the impact of pre-planting soil moisture conditions on the subsequent performance of plants.

### Soil preparation and compost mix

The soil used in this study was a sandy loam, Urrbrae red-brown earth (Alfisol), collected from the 0–10 cm soil layer of The University of Adelaide Waite Campus Arboretum, South Australia. The soil was air-dried and passed through a sieve (<2 mm) to remove any large rocks and coarse woody debris, and mixed with fine sand in a ratio of 7:3 (soil:sand, w/w). To increase the inoculum potential of arbuscular mycorrhizal fungi (AMF) of the soil/sand mixture, pot culture (raised on *Tagetes*) inoculum containing propagules (spores and colonised root fragments) of *Rhizophagus irregularis* (WFVAM23) was added to the mixture in a ratio of 9:1 (soil/sand:inoculum, w/w). The mixture of soil/sand and AM inoculum is referred to as ‘soil’ hereafter.

A compost addition treatment was established (see below) using a commercially-available municipal green-waste compost^37^. The fresh compost was added to the dry soil at a rate of 38.1 g/kg; this rate of application was equivalent to 6 t/ha, assuming all compost was incorporated into the top 10 cm of soil as in earlier studies in a similar system^38^. Physicochemical properties of the un-amended soil, the raw compost, and the compost-amended soil are presented in Table 1.

**Table 1:** Physiochemical properties of un-amended soil, raw compost and compost-amended soil. Values are means (SE), n = 4.

Properties	Raw Compost	Un-amended Soil	Compost-amended soil
pH (1:5)	7.82 (0.04)	6.18 (0.19)	6.41 (0.09)
EC (1:5) (μS. cm^−1^)	3.15 (0.03)	78.90 (1.10)	210.06 (7.09)
Total organic C (mg g^−1^)	298.29 (0.68)	34.09 (0.09)	36.18 (0.02)
Total N (mg g^−1^)	22.94 (0.06)	2.42 (0.01)	2.60 (0.01)
C/N ratio	13.01 (0.01)^(c)^	14.12 (0.02)^(a)^	13.90 (0.05)^(b)^
Ammonium NH_4_^+^-N (mg kg^−1^)	1.53 (0.03)^(c)^	3.48 (0.02)^(b)^	3.72 (0.05)^(a)^
Nitrate NO_3_^−^-N (mg kg^−1^)	957.39 (10.82)^(a)^	7.88 (0.10)^(c)^	50.57 (1.37)^(b)^
Available (Colwell) P (mg kg^−1^)	158.61 (2.18)^(a)^	6.61 (0.21)^(c)^	10.04 (0.29)^(b)^

Means followed by the same letter within rows are not significantly different at the P < 0.05 level (see text).

In order to establish soil-watering treatments (see below) it was first necessary to determine the Water Holding Capacity (WHC) of the soil with and without compost added. This was done by packing the un-amended, and compost-amended, soil into sintered glass funnels connected to a 100 cm water column (ψm = −10 kPa) and to a bulk density 1.36 g/cm^3^, which is the same as the field site from where it was collected. Water was then added to the soil in the funnels in excess allowed to drain for 24 hrs under suction (ψm = −10 kPa). The gravimetric water content of these soils was then determined by drying a soil sample at 105 °C for 48 hrs, and the water loss was expressed as a percentage of soil dry mass. The gravimetric water content of both the un-amended and compost-amended soils at WHC was 28%. The lack of difference in WHC of the compost amended and un-amended soils is like due to the fact that the amount of compost added to the soil was small (see above).

### Experimental design

The experiment was set up with a 2 × 3 × 2 factorial design with two compost addition treatments (with and without compost), three pre-planting soil moisture treatments (wet, dry and cycle), and two plant species (tomato and wheat). Each treatment was replicated five times giving 60 plants in total.

Pre-planting soil moisture treatments were established as follows. To individual plastic bags, 1 kg of soil and 38.1 g fresh compost (for the compost addition treatment) were added, to which Reverse Osmosis (RO) water was applied to give a moisture content of 75% of WHC (wet treatment, 20 pots; cycle treatment, 20 pots) or 25% of WHC (dry treatment, 20 pots). The soils were then packed into plastic, non-draining pots to a bulk density of 1.36 g/cm^3^. All pots were then placed in a glasshouse on the Waite Campus of the University of Adelaide. Conditions in the glasshouse were set at 22 °C day and 17 °C night with supplemental lighting (1000 W metal halide lamps) for a 16/8 hours day/night photoperiod.

**Figure 4:**
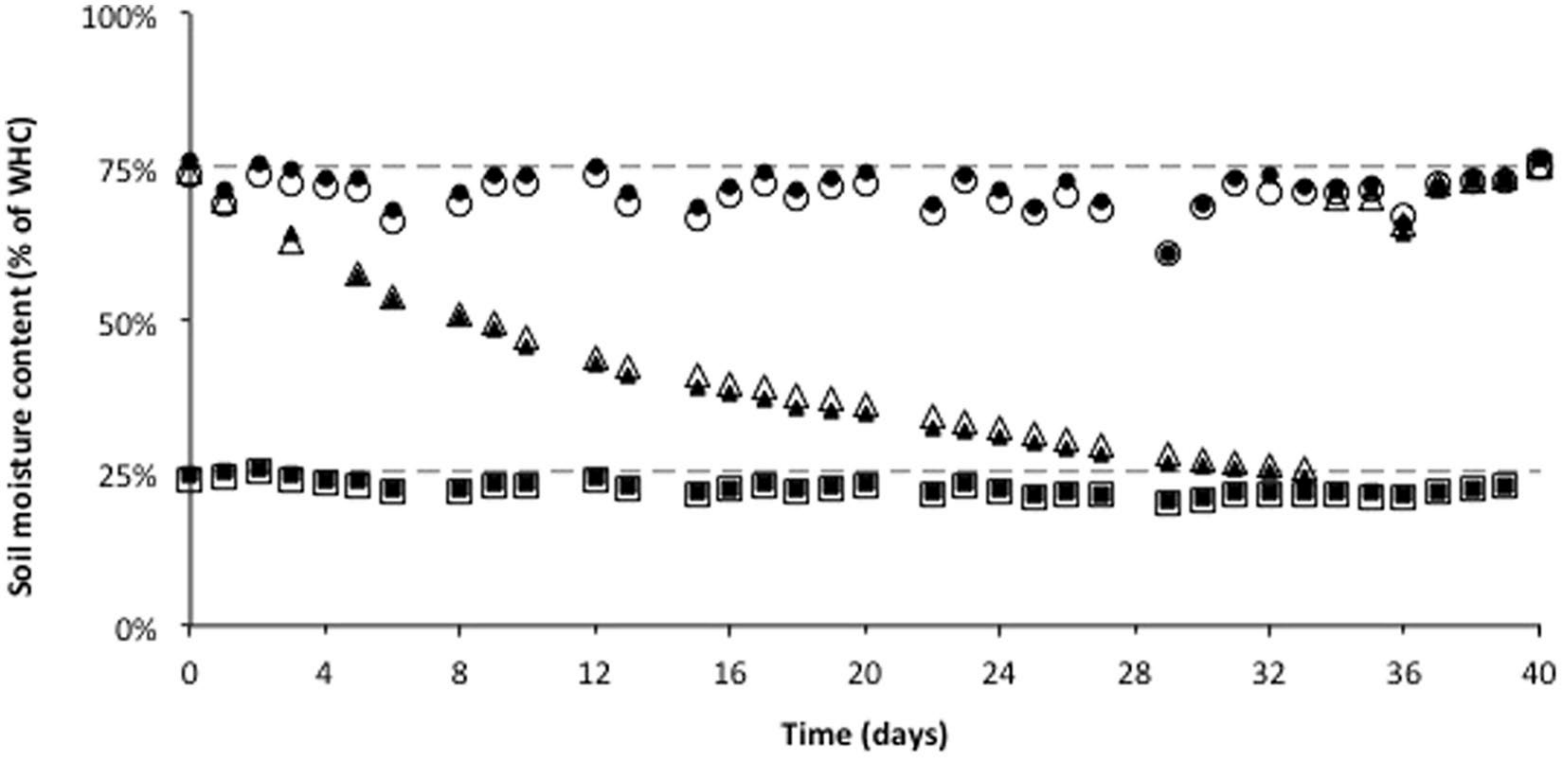
Pre-planting soil moisture conditions. Soil moisture conditions are expressed as a percentage of soil Water Holding Capacity (WHC). Soil that was amended with compost, and un-amended controls are represented by closed and open symbols, respectively. Pre-planting moisture treatments were Wet (circle), Dry (squares) and Cycle (triangles) and described in the text. Values are means, n = 10. Standard errors were extremely small and so are not visible on the figure at this scale, and so are not included. N. B. plants were introduced into these pots on day 40, after which all pots were maintained at 75% of WHC – see text for details.

The experiment involved a pre-planting incubation phase where soils were maintained under different soil moisture conditions (Fig. 4). Soil moisture was monitored by weighing pots on a regular basis (every, or every second, day) and adding water (by weight) as required. The pots in the wet treatment were maintained at 75% of WHC for the duration of the pre-planting phase of the experiment (0–40 days). The pots in the dry treatment were maintained at 25% of WHC for the duration of the pre-planting phase of the experiment (0–40 days). Pots in the cycle treatment, which were at 75% of WHC at the start of the pre-planting incubation phase, were allowed to dry down naturally in the glasshouse until they reached 25% of FC; this was achieved 34 days after commencement of the experiment. On day 35, the pots in the cycle treatment were wet up to 75% of WHC where they were maintained until day 40. Also on day 40, plants in the dry treatment (i.e. 25% of EHC) were wet up to 75% of WHC; thus, all pots in all treatments were at 75% of WHC on day 40.

On day 40 a soil sample was collected from each pot by taking a soil core (approx. 20 g of soil) from the centre of each pot. The soil in the cores was retained for analysis (see below). Into each pot, a single tomato seedling (*Solanum lycopersicum* L., variety 76 R), or wheat seed (*Triticum aestivum* L., variety Axe), was planted/sown. For tomato plants, seeds were surface-sterilized in an aerated 4% sodium hypochlorite (NaClO) solution and pre-germinated on moist filter paper for 5 days in an incubator at 25 °C. The pre-germinated seeds were then sown in individual seed raising containers, each containing approximately 50 g of sterile sand. The tomato seedlings were grown in this substrate for 3 weeks before transplanting into the experimental pots. For wheat, seeds were surface-sterilized and pre-germinated one day before transplanting into the experimental pots. The soil removed from each pot by coring at the time of planting was replaced with an equivalent mass of coarse-fine sand mix^10^. Immediately after transplanting, a layer of plastic beads was placed on the soil surface to limit evaporation, and all pots were maintained at 75% of WHC by weight for the remainder of the experiment.

### Soil analysis, and plant harvesting and analysis

Soil samples collected from each pot at the time of planting were analysed as follows. Concentrations of ammonium (NH_4_^+^ - N) and nitrate (NO_3_^−^-N, plus nitrite) were measured colorimetrically on duplicate 2 M KCl extracts (1:2.5 soil: extractant ratio) following a modification of Miranda, *et al*.^39^ and Forster^40^, respectively. Gravimetric soil moisture content was determined as described above. All remaining soil was dried at 40 °C for 24 hours. Plant available (Colwell) P was extracted in 0.5 M sodium bicarbonate (1:10 soil:extractant ratio), and P concentration measured colorimetrically following the method of Murphy and Riley^41^.

All plants were harvested 48 days after planting. In other words, plants were harvested 88 days after the start of the experiment; thus, there was a 40 day pre-planting incubation phase where soil moisture was varied, and 48 days of plant growth phase where soil moisture was held constant (see above and Fig. 4). At the time of harvest, plant above-ground biomass was cut at the soil surface and shoot fresh weights for tomato, and shoot and head fresh weights for wheat, recorded. The plant roots were gently washed from the posts with reverse osmosis (RO) water to remove soil, and root fresh weights measured. A weighed sub-sample of roots (0.1 g for wheat and 0.2 g for tomato) was cleared and stained with ink and vinegar^42^ and mycorrhizal colonisation measured using the grid-line intersect method^43^. All of the shoots, heads and remaining root biomass where oven-dried at 60 °C until a constant weight was achieved, and dry weights measured. The dry plant samples were then ground to a fine powder using a pulverising mill before measuring total N and total P as follows. Total N was measured using the Dumas method by APAL services (http://www.apal.com.au/; last accessed April, 2017). For total P, plant tissues were digested in a mixture of concentrated nitric acid (HNO_3_) and hydrogen peroxide (H_2_O_2_, 30%) (4:1, v/v) in closed 50 mL polypropylene tubes in a heat block. The block was set with two cycles. The first cycle was 80 °C for 30 minutes and the second cycle was 125 °C for 150 minutes. Total P in the digests was measured by the modified phosphovanado-molybdate complex method^44^.

### Statistical analysis

Data were analysed by two-way analysis of variance (ANOVA) using general linear model (GLM). The factors in the analysis were pre-planting moisture conditions and compost addition. For soil properties prior to planting, there were 10 replicates for each treatment combination. Plant data at harvest (biomass, nutrient contents and concentrations, and mycorrhizal colonisation) were analysed separately for tomato and wheat; thus, there were five replicates for each treatment combination for each species. Pairwise comparisons were performed using Tukey’s HSD tests. All data analyses were performed in the R statistical environment version 3.3.1 (2016-06-21), using the ‘agricolae’ and ‘car’ packages.
